# Serum Sialic Acid as a Biomarker of Inflammation and Infection: Insights From Veterinary Medicine

**DOI:** 10.1155/vmi/4769993

**Published:** 2026-05-14

**Authors:** Tina Yaghoobpour, Milad Faraji, Saeed Nazifi

**Affiliations:** ^1^ Department of Clinical Sciences, School of Veterinary Medicine, Shiraz University, Shiraz, Iran, shirazu.ac.ir; ^2^ Department of Basic Sciences, School of Veterinary Medicine, Shiraz University, Shiraz, Iran, shirazu.ac.ir

**Keywords:** biomarker, lipid-bound sialic acid, protein-bound sialic acid, total sialic acid, veterinary medicine

## Abstract

Serum sialic acid (SSA) levels, including total sialic acid (TSA), lipid‐bound sialic acid (LBSA), and protein‐bound sialic acid (PBSA), have been extensively studied as biomarkers of inflammation and infection across various species and diseases. In parasitemic sheep, elevated SSA levels likely reflect host–pathogen interactions and immune activation. Contrasting findings in bovine theileriosis demonstrated increased sialic acid (SA) levels during acute infections but decreased levels in severe parasitemia, suggesting variable responses based on parasitemia rates. SSA and LBSA levels in dogs significantly increase in babesiosis and dirofilariasis, indicating tissue damage and oxidative stress. Babesiosis, a tick‐borne infection, shows SA elevations associated with inflammation and red blood cell destruction. Dirofilariasis, a heartworm disease, shows elevated SSA and LBSA levels, highlighting the importance of acute‐phase proteins in disease severity. In avian species, diseases like Newcastle disease virus (NDV) infection, infectious bronchitis virus (IBV) infection, and bacterial challenges caused significant increases in SSA, LBSA, and PBSA levels, with LBSA showing the most pronounced changes, indicating its high sensitivity to inflammatory processes. Japanese quail with omphalitis also demonstrated elevated LBSA, highlighting its diagnostic potential in avian inflammation. Cattle with conditions such as traumatic reticuloperitonitis, acute metritis, pneumonia, and babesiosis exhibited markedly elevated SA and APP levels, consistent with findings in mastitis, colisepticemia, and leptospirosis, where APP levels correlated with disease severity. Tissue damage associated with increased SA is closely linked to inflammation and infection. These changes may also influence receptor–ligand interactions during infection, supporting SA as a useful biomarker for disease monitoring.

## 1. Introduction

Sialic acids (SAs) are nine‐carbon monosaccharides located at the nonreducing ends of glycoproteins and glycolipids. They often occupy exposed termini of glycoconjugates and act as ligands for siglecs and selectins, which mediate key cell–cell adhesion events in inflammation and immunity [1]. Galectins and siglecs are also important mediator families that participate in cellular interactions during inflammation and immune regulation [1, 2]. Collectively, these molecular interactions provide a mechanistic basis for why alterations in serum sialic acid (SSA) may reflect inflammatory activity in animal diseases.

Taniuchi et al. [3] suggested that lipid‐bound sialic acid (LBSA) in serum could serve as a marker of inflammatory disorders and the acute‐phase response. Rosenberg and Schengrund [4] reported that many acute‐phase proteins (APPs), including orosomucoid, antitrypsin, haptoglobin (Hp), ceruloplasmin (Cp), fibrinogen, complement proteins, and transferrin, carry sialylated termini on their oligosaccharide chains. Several mechanisms have been proposed to explain increased SSA concentrations, including increased sialylation of serum proteins, decreased desialylation of plasma glycoproteins, and an increase in acute‐phase reactants containing SA [5]. Since activation of the acute‐phase response is a conserved mechanism across domestic animal species, these changes are clinically relevant in veterinary inflammatory and infectious diseases [6].

Circulating SA is mainly present as protein‐bound sialic acid (PBSA) and LBSA; total sialic acid (TSA) represents the sum of these fractions [7]. Since SA is a key component of membrane structure and is present at the terminal ends of several APPs, SSA concentrations may increase in association with cellular and tissue damage [8]. Such changes can provide useful diagnostic and prognostic information [9]. SSA may also rise with erythrocyte membrane destruction [10], and 70a strong association between SSA levels and parasitemia rates has been demonstrated in animals infected with hemoprotozoan parasites [8]. Therefore, in veterinary inflammatory and infectious diseases, particularly hemoparasitic disorders, measurement of SSA and its fractions may provide supportive information on inflammatory burden and tissue injury.

Receptor availability is also a critical determinant of virus–host range [11]. Influenza A viruses (IAVs) attach to host cells via SA receptors, and avian‐origin viruses preferentially bind *α*2,3‐linked SAs, whereas classical swine and human IAV strains preferentially bind *α*2,6‐linked SAs [11–13]. These host‐related differences in SA receptor linkage patterns have implications for interspecies transmission and tissue tropism, which are key considerations for surveillance and control of viral diseases in animals.

Beyond infection and inflammation, elevated SSA has been reported in association with increased cancer risk [14, 15], including correlations with prostate cancer [16, 17] and clinical indicators of disease aggressiveness [18]. While many of these early observations originated from human clinical research, similar inflammatory and membrane‐associated mechanisms have subsequently been documented across veterinary species.

Tissue distribution and linkage‐specific detection of SAs can be investigated using lectins: *Sambucus nigra agglutinin* (SNA) is commonly used to detect *α*2,6‐linked SAs, while *Maackia amurensis lectins* (MAL‐I and MAL‐II) are used to detect *α*2,3‐linked SAs in specific contexts [19, 20]. N‐acetylneuraminic acid (NANA), a common SA form on cell membranes, has been linked to inflammatory disorders and endothelial dysfunction, suggesting that elevated serum levels may reflect membrane injury, particularly in vascular tissues.

The epithelial lining of the upper airways is a primary site of *α*2,6‐linked SA receptor expression in animals, consistent with receptor preferences of IAV strains originating from humans and pigs [21]. Animals synthesize SAs, and Neu5Ac and Neu5Gc are the two most prevalent forms [22, 23]. Although IAVs bind to both, Neu5Gc appears to lack a significant functional role in this context [24, 25]. Importantly, in veterinary medicine, species, age, production stage, and physiological status should be considered when interpreting SSA concentrations. SAs are also present in milk, and Neu5Ac levels vary across lactation in cows and goats [26, 27], emphasizing that physiological state and species‐related factors should be considered when interpreting SA measurements in veterinary settings.

Modern analytical technologies, including biosensor‐based approaches, mass spectrometry, and high‐performance liquid chromatography, have improved SA detection and structural characterization [28–30]. However, high cost and technical complexity remain important limitations. Ishigaki and Itoh [31] described a flow cytometry‐based method to detect *α*2,3‐ and *α*2,6‐linked SAs on cell surfaces using biotinylated MAL‐II and SNA, with staining specificity confirmed by sialidase treatment. Collectively, these methodological advances support more accurate evaluation of SA patterns, while serum‐based measurements remain particularly relevant for practical biomarker use.

While the molecular and clinical foundations of SA research were originally developed in human medicine, mounting evidence from livestock and companion animals underscores its rising significance in veterinary diagnostics.

Nonetheless, despite substantial evidence associating SSA with inflammatory, viral, metabolic, and neoplastic disorders, its clinical interpretation remains variable between species, illness classifications, and analytical techniques. Moreover, new findings regarding SA receptor biology, host–pathogen interactions, and advancements in analytical technologies require a reassessment of SSA as a biomarker in both human and veterinary medicine. A thorough and current assessment of SA dynamics in many animal diseases is necessary to elucidate its diagnostic, prognostic, and pathophysiological importance.

To address this need, the present review evaluates SA concentrations in various diseases across different animal species.

## 2. Literature Search Strategy

This narrative review was derived from a systematic literature search performed in PubMed, Scopus, Web of Science, and Google Scholar for research published till 2025. The search contained combinations of terms like “sialic acid,” “serum sialic acid,” “acute phase response,” “inflammation,” “infectious diseases,” “biomarkers,” and “animals,” utilizing Boolean operators (AND, OR).

Included were peer‐reviewed literature published in English relevant to inflammatory, infectious, metabolic, and neoplastic disorders in both animals and humans. Recent and mechanistic investigations were prioritized, although foundational earlier publications were referenced where needed. Reference lists of chosen papers were examined to discover further pertinent studies.

### 2.1. Large Ruminants

These findings suggest that, in large ruminants, SSA elevation mainly reflects acute‐phase activation and membrane injury rather than disease‐specific mechanisms [32]. Serum SSA and LBSA levels were consistently raised in the majority of these diseases, indicating a connection with inflammatory activation. Similarly, Deger et al. [33] found significantly higher serum concentrations of LBSA and SSA in bovine babesiosis, and subsequent investigations confirmed large increases in SSA and LBSA in calves infected with blood parasites such as *Theileria* spp. and *Anaplasma* spp. [10, 34]. Overall, our findings point to a typical pattern of the SSA rise in bovine infectious and hemoparasitic illnesses.

However, not all studies produce uniform outcomes. Yurtseven and Uysal [35] found a substantial drop in SSA levels in calves with severe parasitemia (50%–70% induced by *T. annulata*). Differences in parasitemia rates and disease stage (acute versus chronic theileriosis) could explain these discordant results. This implies that SSA dynamics in cattle may vary depending on infection severity and parasite burden, emphasizing the significance of contextual interpretation.

These findings suggest that in large ruminants, SSA elevation primarily reflects acute‐phase activation combined with membrane injury rather than disease‐specific mechanisms [32]. Although the precise mechanism remains unclear, SSA correlates with major APPs such as alpha‐1 acid glycoprotein (AGP) [3, 5]. Increased production of sialylated acute‐phase glycoproteins can raise circulating SA, since acute‐phase reactants influence TSA concentrations due to their glycoprotein structure [36]. In addition, membrane damage may release SA–containing glycoproteins and glycolipids, increasing SSA and LBSA [5]. This mechanism may also explain the elevated TSA levels observed in cattle with botulism, where neuromuscular paralysis and systemic intoxication were associated with significant increases in SSA [37].

Studies on the link between SA and important APPs provide new information. Nazifi [38] found a positive link between LBSA and PBSA in clinical mastitis but a negative correlation in subclinical mastitis, implying that illness stage affects biomarker behavior. Although SAA and Hp levels were significantly higher in ill cattle compared to healthy animals, they did not reliably correlate with one another or with any evaluated factors [38]. Nonetheless, several studies have shown that both SAA and Hp levels rise significantly after tissue injury [39, 40] and that their concentrations predict the severity of inflammation [41–44]. These constant rises across investigations increase the reliability of combining SSA fractions with major APPs to identify healthy from ill cattle.

Further evidence from foot‐and‐mouth disease (FMD) research backs up this integrative approach. Infected cattle showed significant relationships with PBSA, SSA, TNF‐α, and Cp [45], highlighting the complicated relationship between cytokines and acute‐phase responses. Uzlu et al. [46] found that FMD altered both SA and oxidative stress parameters, with reduced glutathione levels much lower in infected bulls. Similarly, in bovine leptospirosis, infected animals had higher SSA, LBSA, malondialdehyde (MDA), and nitric oxide (NO) levels while having lower antioxidant capacity [47]. These findings collectively indicate that high SSA reflects not only APP synthesis, but also oxidative stress and membrane damage.

**FIGURE 1:**
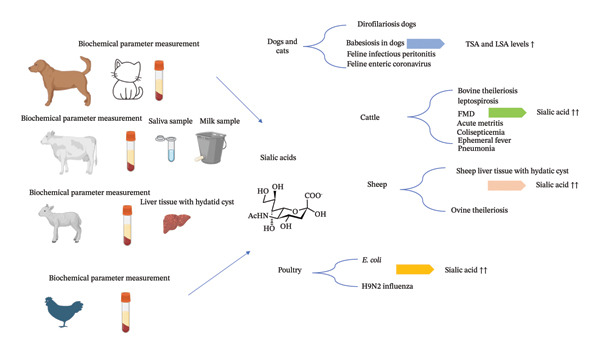
Serum sialic acid as a biomarker of inflammation and infection in domestic animals.

The majority of bovine studies indicate consistent elevations in SSA and LBSA under inflammatory and infectious conditions. Variability arises notably in instances of severe parasitemia, where the intensity of sickness may influence SA dynamics. The evidence indicates that SSA, specifically SSA and LBSA, serves as a dependable biomarker for inflammation in cattle, especially when correlated with notable APPs and oxidative stress indicators (Figure 1) (Table 1).

**TABLE 1:** Changes in serum sialic acids as a biomarker of inflammation and infection in various diseases.

Authors	Disease	Species	Results
Nazifi et al., 2009)	Acute local traumatic reticuloperitonitis	Bovine	TSA and PBSA‗TRP, pneumonia, and metritis

Nazifi et al., 2011	Clinical and subclinical mastitis	Bovine	Protein‐bound sialic acid does not change in subclinical mastitis LBSA and PBSA‗clinical mastitis LBSA and PBSA ≠ subclinical mastitis

Nazifi et al., 2012	FMD‐infected	Bovine	TNF‐α, INF‐γ, and sialic acids and acute‐phase proteins (SAA, Hp, ceruloplasmin, and fibrinogen)↑↑ in FMD‐infected cattle

Karagenc et al., 2005	Theileriosis	Bovine	Serum sialic acid concentrations↑

Erdogan et al., 2008	Leptospirosis	Bovine	TSA, LBSA, MDA, NO, UA, TP, glucose ↑ GSH and albumin ⬇

Uzlu et al., 2016	Foot‐and‐mouth disease	Bovine	Serum and saliva GSH ⬇ Serum and saliva total sialic acid (SA), malondialdehyde (MDA), and nitric oxide (NO)↑

Kızıltepe et al., 2024	Liver tissue with a hydatid cyst	Ovine	NO, MDA, Cp, and TSA in the ↑↑ GSH levels ⬇

Razavi et al., 2010	Theileriosis	Ovine	TNF‐α, IFN‐γ, and sialic acid ↑↑

Nazifi et al., 2011	Infectious bronchitis virus–infected chicks	Chicks	TSA, LBSA, PBSA, Hp, and SAA ↑ l LBSA ≥ obvious change

Ghaemmagham et al., 2021	Live vaccine virus and a virulent Newcastle disease Virus	Chicks	Challenge with NDV inflammatory factors (haptoglobin, serum amyloid A, ovotransferrin, adenosine deaminase, serum proteins, total protein, albumin, globulins), and gangliosides (total sialic acid, lipid‐bound sialic acid, and protein‐bound sialic acid) and acute‐phase response↑↑↑ vaccination ↑, these parameters

Dadras et al., 2014	*E. coli* O2 and H9N2 influenza	Broiler chickens	Serum sialic acids ↑, avian influenza virus, and *E. coli* O

Manzari Tavakoli et al., 2020	Induced by *Escherichia coli* Lipopolysaccharide in Broiler Chickens	Broiler Chickens	Meloxicam and dexamethasone lipid‐bound sialic acid and protein‐bound sialic acid,⬇ dexamethasone ⬇ total sialic acid levels

Kesici et al,2020	Dirofilariasis	Dogs	TSA and LSA levels↑

Ozdek et al., 2023	Babesiosis	Dogs	TSA and LSA levels. Serum AST, ALP, LDH, and CK enzyme activities and CRP, glucose, globulin, total bilirubin, urea, uric acid, creatinine, and BUN ↑↑ Total protein ⬇

Rossi et al., 2009	Feline infectious peritonitis	Feline	TSA ↑

Desmarets et al., 2014	Feline enteric coronavirus	Feline	FECV can bind sialic acids to facilitate enterocyte infection

### 2.2. Small Ruminants

Razavi et al. [48] found that sheep with various parasitemia levels in malignant ovine theileriosis had significantly higher SSA fractions (SSA, LBSA, and PBSA). Similar increases in blood SA have been observed in bovine theileriosis [34], indicating that SA elevation is a general response to hemoprotozoan infections in all ruminant species. Karagenc et al. [34] found that *Theileria annulata* infection in acute tropical theileriosis is associated with elevated SSA levels, confirming the relationship between parasite‐induced inflammation and SA dynamics.

Yurtseven and Uysal [35] reported opposing findings, showing considerably lower SSA levels in calves with severe parasitemia (50%–70%) caused by *Theileria annulata*. These disparities could be related to variations in parasitemia intensity and illness stage. While moderate parasitic infection appears to induce acute‐phase activation and membrane disruption, resulting in increased circulating SA, severe parasitemia may cause consumption, receptor‐mediated binding, or SSA depletion. Thus, parasitemia load is likely a key predictor of SA behavior in hemoprotozoan illnesses.

Elevated SA levels may alter cell‐to‐cell and host–parasite interactions. SA can mask lactosaminic sequences, preventing them from interacting with SA‐binding lectins [49] or galactose‐specific lectins [50]. This masking effect may change receptor–ligand interactions during infection, potentially influencing parasite adherence and invasion. Conversely, cellular breakdown during inflammation causes the release of SA‐containing glycolipids and glycoproteins into the bloodstream [51], which contributes to elevated serum SSA levels. Early‐stage glycoprotein changes in ovine theileriosis may activate immune‐mediated sialylation mechanisms, which can accelerate APP production and inflammatory responses [51].

Parasitic illnesses in sheep provide additional evidence for the relationship between SA, oxidative stress, and tissue injury. In cystic echinococcosis, Kızıltepe et al. [52] found elevated levels of SSA, NO, MDA, and Cp in infected sheep, as well as lower levels of reduced glutathione. These data suggest that elevated SSA is intimately linked to oxidative stress and hepatic tissue damage. Overall, investigations on ovine parasitic illnesses show a consistent pattern of higher SSA in conjunction with inflammatory activation and oxidative imbalance; severe parasitemia may alter this response.

Current evidence indicates that SSA fractions in sheep serve as effective indicators of inflammation and parasitic load. Consensus among several studies corroborates the reliability of SSA elevation in moderate sickness, but observed discrepancies highlight the need to interpret SSA results concerning parasitemia intensity and disease stage (Figure 1) (Table 1).

### 2.3. Companion Animals (Dogs and Cats)

According to Kesici et al. [53], dogs infected with dirofilariasis had considerably higher serum SSA levels than healthy controls. This increase is most likely caused by increased production of APPs, which have terminal SA residues on their oligosaccharide chains. Carretón et al. [54] found elevated APP levels in dogs with dirofilariasis, implying that cardiopulmonary impairment and tissue injury promote acute‐phase activation. In addition to SSA, Kesici et al. [53] found considerably higher levels of LBSA in infected dogs. Given that 85%–90% of circulating SA is protein‐bound and 10%–15% is lipid‐associated, alterations in certain fractions may represent different pathophysiological processes.

Although increased APP synthesis largely affects the protein‐bound portion, dirofilariasis has also been linked to higher lipoprotein‐related SA levels, probably due to increased hepatic lipoprotein production. Notably, affected dogs had elevated VLDL and LDL levels, with LDL above standard limits. Furthermore, oxidative stress and membrane injury may contribute to increased SSA and LBSA by releasing SA residues from damaged cellular membranes [55]. Overall, these findings imply that SSA rise in canine dirofilariasis reflects both acute‐phase activation and changes in lipid metabolism, bolstering its utility as a supportive inflammatory biomarker.

Furlanello et al. [56] described significant systemic consequences associated with canine babesiosis caused by erythrocyte destruction, culminating in multiorgan failure. Erkilic [57] emphasized that inflammation and hemolytic anemia are central to the clinical presentation of babesiosis. This supports the interpretation that hemolysis‐driven membrane disruption is a key determinant of SSA elevation in canine babesiosis.

Hemolysis may cause an increase in circulating SA levels beyond membrane disruption. Protozoan parasites are known to interact with sialylated surface molecules during host invasion. Similar to the malaria parasite *Plasmodium falciparum*, which utilizes SA‐dependent pathways for erythrocyte recognition, hemoprotozoan infections may alter sialylation patterns and contribute to circulating SSA changes. Thus, increased SSA in canine babesiosis may reflect both erythrocyte destruction and infection‐related modulation of sialylated glycoconjugates [58].

Consistent with this approach, greater SSA concentrations were seen in dogs infected with *Babesia canis canis* [59], and infected animals had considerably higher SSA and LBSA levels than healthy controls [60]. Similar increases in SA levels have been seen in other species infected with *Babesia* [8, 10, 33, 61], demonstrating cross‐species agreement on the inflammatory and hemolytic mechanisms behind SA elevation.

Taken together, dog studies show continuous increases in SSA and usually LBSA in both dirofilariasis and babesiosis. The consistency of results across separate studies increases the credibility of SSA as a supporting marker of inflammatory and hemolytic load in canine parasite illnesses. The proportional contribution of protein‐bound vs lipid‐bound fractions may vary depending on the degree of acute‐phase activation, lipid metabolism alterations, or membrane breakdown.

In feline infectious illnesses, SA appears to contribute to viral pathogenesis. SA‐binding capacity has been established by feline enteric coronavirus (FECV), albeit it is not strictly essential for infection onset [62]. In feline infectious peritonitis (FIP), the combined SSA and AGP test had low diagnostic accuracy, correctly identifying only around 60% of true positives and true negatives [63]. These data show that, while SSA helps us understand viral–host interactions, its diagnostic specificity in cats may be restricted when used alone. As a result, similar to canine infections, SSA in feline disorders is most useful when read in conjunction with other inflammatory indicators rather than as a solo diagnostic test.

SSA levels in dogs and cats are regularly raised during parasitic and inflammatory disorders, especially those associated with hemolysis and acute‐phase response. The diagnostic reliability is enhanced when SA assays are integrated with validated biomarkers and clinical observations (Figure 1) (Table 1).

### 2.4. Poultry

Previous studies have repeatedly shown that blood indicators such as SSA, LBSA, PBSA, Hp, and SAA are considerably higher in ill birds compared to healthy controls. Nazifi et al. [64] investigated these biomarkers in chicks infected with infectious bronchitis virus (IBV) and discovered significant increases in LBSA, PBSA, SAA, and Hp. These findings are consistent with previous observations [10, 65–67], which show a repeatable pattern of SA and APP increase during IBV‐associated inflammation. Notably, LBSA showed the most pronounced change, indicating more sensitivity than the other fractions. This repeated finding across investigations adds to the dependability of LBSA as a responsive biomarker of inflammatory processes in poultry.

IBV‐induced diseases such as tracheitis, bronchitis, tracheal edema, bronchial caseous plugs, and air sacculitis cause acute‐phase reactions with increased SAA and Hp production [64]. SSA levels rise rapidly after an inflammatory injury [5], and pathological factors such as tissue destruction and proliferation lead to elevated circulating SA levels [68]. Cytokine‐mediated stimulation of the acute‐phase response promotes hepatic sialylated glycoprotein production, increasing serum SSA levels [69]. In the IBV model, the simultaneous rise of SA fractions with SAA and Hp shows that serum SA reflects inflammatory load and tissue injury in avian viral illness.

Similar trends are seen in other avian inflammatory diseases. Significant increases in acute‐phase reactants, inflammatory mediators, and gangliosides, such as SSA, LBSA, and PBSA, have been seen in Japanese quails with retained yolk sac [70]. Again, LBSA showed the most significant change, indicating its potential as a sensitive biomarker in avian inflammation. This consensus across independent avian investigations boosts confidence in the diagnostic use of LBSA.

Experimental NDV and LPS challenge models consistently demonstrate rapid acute‐phase activation accompanied by SSA elevation [71, 72]. These data indicate the strong relationship between cytokine signaling (IL‐1, IL‐6, TNF‐α) and APP synthesis in birds, as previously observed [64, 73]. Elevated SAA and Cp levels during influenza infection demonstrate that APPs are early indications of viral illness in avian species [74].

From a molecular standpoint, influenza A viral receptor specificity helps to understand host vulnerability. Avian IAVs preferentially bind *α*2,3‐linked SA receptors [11, 75, 76], while human and swine strains prefer *α*2,6 links [12, 77]. The presence of *α*2,3‐linked receptors in avian respiratory and intestinal organs explains viral tropism and pathogenicity. Dadras et al. [78] found that coinfection with the H9N2 influenza virus and *Escherichia coli* O2 caused significant changes in inflammatory mediators and gangliosides, with SSA concentrations increasing over time. This indicates that SSA may reflect inflammatory synergy during coinfections.

In several viral, bacterial, and experimental inflammatory scenarios in avians, there is a consensus that serum SSA and, particularly, LBSA levels consistently increase in response to inflammatory activation. Although SAA serves as a rapid and sensitive acute‐phase marker, substantial data indicate that LBSA may possess further discriminatory value. The magnitude of the differences seems to be influenced by the severity of the infection, the timing, and the immunological status. When assessed with established APPs, the current findings endorse SSA, especially LBSA, as a reliable marker of inflammatory activity in avian medicine (Figure 1) (Table 1).

### 2.5. Comparative Interpretation Across Species

In cattle, small ruminants, companion animals, and poultry, a continuous trend is observed in which SSA and LBSA increase in response to inflammatory and infectious stimuli. This interspecies agreement enhances the biological validity of SSA as a universal marker of acute‐phase activation.

Discrepancies are noted in instances of severe parasitemia, especially in hemoprotozoan infections, where SSA levels may diminish. The variations indicate that parasite load, illness progression, and membrane integrity affect SA dynamics.

Significantly, LBSA exhibits heightened sensitivity in avian inflammatory models, but in ruminants and canines, both SSA and LBSA consistently present elevated levels. Diagnostic reliability across species is enhanced when SSA fractions are analyzed in conjunction with established APPs like SAA and Hp.

Cross‐species evidence collectively suggests that SSA serves as a supporting inflammatory biomarker rather than a disease‐specific indicator. The primary clinical significance is in integrated biomarker panels rather than isolated measurements.

### 2.6. Diagnostic Value and Clinical Interpretation

Although SSA (LBSA and PBSA) levels are consistently elevated in inflammatory and infectious diseases across different species, their clinical relevance must be evaluated alongside established acute‐phase biomarkers such as serum amyloid A (SAA), Hp, and C‐reactive protein (CRP).

SAA is generally the most sensitive and rapidly responding APP, while Hp is a recognized marker of systemic inflammation, particularly in ruminants. Conversely, SSA reflects various processes, including acute‐phase glycoprotein synthesis, membrane damage, and oxidative stress. The multifactorial origin increases sensitivity for detecting inflammatory activation but limits disease specificity, as elevations occur in diverse clinical contexts.

Significantly, there is a paucity of studies evaluating diagnostic criteria such as sensitivity, specificity, or ROC‐based accuracy for SA fractions, highlighting a considerable research gap. Future studies should determine species‐specific cutoff values and directly assess diagnostic effectiveness relative to SAA, Hp, and CRP. Numerous constraints must be recognized. The majority of research is cross‐sectional, features very limited sample sizes, and lacks standardized analytical methodologies. Interlaboratory heterogeneity may also limit comparability across studies.

Now, SSAs, particularly SSA and LBSA, are optimally understood as supportive biomarkers within a multimarker diagnostic framework rather than as independent diagnostic tests.

## 3. Conclusion

In veterinary medicine, SSA (LBSA and PBSA) and main APPs (Hp and SAA) serve as helpful supporting indicators of infection and inflammation. Increased SA levels in bacterial, viral, and parasite illnesses are indicative of tissue damage, oxidative stress, and acute‐phase activation.

Nevertheless, standardized reference ranges and validation studies appropriate to individual species are still missing. Consistent clinical interpretation is further limited by variability associated with disease stage, parasitemia severity, and analytical techniques.

SSA should not be utilized as a stand‐alone test in clinical settings, but rather as a component of a biomarker panel. Establishing diagnostic cutoffs, assessing prognostic value through longitudinal studies, and creating affordable assays appropriate for standard veterinary practice should be the main goals of future research.

**Nomenclature**
SA: Sialic acid
APPs: Acute‐phase proteins
PBSA: Protein‐bound sialic acid
LBSA: Lipid‐bound sialic acid
TSA: Total sialic acid
MAL‐I: *Maackia amurensis lectin-I*
NANA: N‐acetylneuraminic acid
NDV: Newcastle disease virus
IBV: Infectious bronchitis virus
FMD: Foot‐and‐mouth disease
Hp: Haptoglobin
SAA: Serum amyloid A
TNF: Tumor necrosis factor
IL‐6: Interleukin 6
GSH: Reduced glutathione
MDA: Malondialdehyde
NO: Nitric oxide

## Author Contributions

Saeed Nazifi: designing the review article and writing–review and editing.

Tina Yaghoobpour: original draft writing–review and editing.

Milad Faraji: writing–review and editing.

## Funding

No funding was obtained for this study.

## Disclosure

All authors have seen and approved the final version of the manuscript being submitted.

## Ethics Statement

This study was conducted under the supervision of the Iranian Society for the Prevention of Animal Cruelty and the Research Council of Shiraz University (IACUC No. 6387/63).

## Conflicts of Interest

The authors declare no conflicts of interest.

## Data Availability

The datasets used and/or analyzed during the current study are available from the corresponding author upon reasonable request.
